# The Impact of Internet Use on the Social Networks of the Elderly in China—The Mediating Effect of Social Participation

**DOI:** 10.3390/ijerph19159576

**Published:** 2022-08-04

**Authors:** Qunlin Zhang, Zhibin Li

**Affiliations:** School of Management, Xi’an Polytechnic University, Xi’an 710048, China

**Keywords:** internet use, older adults, social networks, social participation

## Abstract

Introduction: Under the overlapping interaction of digitization and aging, the number of elderly Internet users has increased yearly. However, the impact of Internet use on the social networks of the elderly is still unclear. Methodology: In this study, we utilize the methods of ordinary least square regression (OLS), propensity score matching (PSM), instrumental variable (IV), and Bootstrap-mediated effect analysis methods using data from the Chinese General Social Survey (CGSS) to analyze the impact of Internet use on the social networks of the elderly and examine the mediating effect of social participation. Objectives: A total of 1363 validated respondents aged 60 or above were included to explore the relationship between Internet use, social networks, and social participation among the elderly in China. Results: The results show that Internet use positively and significantly impacts the social networks of the elderly. Compared to the elderly who do not use the Internet, the elderly who use the Internet have a larger social network size, more significant social network heterogeneity, and higher social network upper reachability. The mediated analysis shows that social participation plays a positive mediating role in the influence of Internet use on the social networks of the elderly. That is, Internet use will benefit the social network of the elderly by improving the level of their social participation. Besides, there also exists heterogeneity in the effect of Internet use on social networks among the elderly with different genders, ages, and places of residence. Conclusions: Internet use benefits the social network of the elderly, and social participation partially mediates the relationship between Internet use and the social network of the elderly. These findings have implications for formulating public policies aimed at active aging; it is necessary to bridge the “digital divide” and promote the digital integration of the elderly. Let more older adults benefit from Internet use, thus improving the social network and quality of life of the elderly.

## 1. Introduction

At present, China is in the social transformation period where informatization, digitalization and aging overlap, and more and more older adults begin to surf the Internet in various ways. According to the data of the 48th Statistical Report on Internet Development in China released by CNNIC (China Internet Network Information Center), the number of Internet users in China has reached 1.011 billion, of which 12.2% are Internet users over 60 years old, and the proportion is increasing year by year. The Internet is gradually integrated into all aspects of the daily life of the elderly and plays an increasingly important role in their later years. The living conditions of the elderly in the digital age have also become a hot-debated topic in the world.

A social network is a collection of relationships between people. As an interpersonal link, a social network links individuals with other network members and realizes the reciprocity or mutual transmission of resources. For the elderly, the informal social support resources carried by social networks will affect their physical and mental health, happiness, and life satisfaction [1,2,3]. As a non-institutionalized social resource, the social network also provides the elderly with unique old-age security functions such as material security, safe security, and spiritual security, which is an essential source for the elderly in China to obtain informal social support and living security in their later years [4,5]. At the same time, social networks also play a pivotal role in promoting the process of active aging [6]. Therefore, under the dual background of the overlapping intersection of digitalization and aging, it is of both theoretical and practical significance to analyze the impact of Internet use on the social network of the elderly in China and its influence mechanism for promoting the realization of active aging.

In China, empirical research on Internet use and the social network of the elderly is relatively scarce. The related study mainly investigates the impact of Internet use on life satisfaction, social adaptation, and the health of the elderly [7,8]. However, the foreign research on this aspect is relatively abundant and mainly forms two completely different viewpoints. One view is that the use of the Internet by the elderly will enhance many aspects of their social lives, such as building new personal friendships, increasing the quantity and quality of contact with family and friends, and expanding social networks [9,10,11]. The other view is that the decrease in Internet use and time spent with friends is significantly related to the decline in local social networks [12]. The use of the Internet will replace face-to-face contact with weaker network connections and reduce the participation of the elderly in social activities [13], thus further shrinking the social network of the elderly [14]. It can be seen that the impact of Internet use on the social network of the elderly needs further study.

Therefore, this paper aims to use the data of CGSS 2017 to explore the influence of Internet use on the social network of the elderly and its influence mechanism and tries to expand the existing research in the following aspects: First, although the current studies have discussed the influence of Internet use on the social networks of the elderly, most of them are based on the western cultural background, and there are significant differences between Chinese and western cultures, so the applicability of some conclusions needs to be discussed. Therefore, this paper will focus on the impact of Internet use on the social network of the Chinese elderly under the Chinese cultural background. Secondly, most studies do not further divide the social networks into different dimensions for in-depth exploration but only analyze them as a holistic variable. Therefore, this paper will deeply explore the influence of Internet use on the social network size, social network heterogeneity and the upper reachability of social networks of the elderly. Thirdly, the current studies mainly examine the direct impact of Internet use on the social network of the elderly and seldom discuss the influence mechanism. Therefore, this paper will try to determine whether social participation plays a mediating role in the relationship between Internet use and the social network of the elderly. Fourthly, in the few empirical studies on Internet use and social network of the elderly in China, most of them do not consider the sample self-selection effect and the endogenous problems caused by the possible two-oriented causal relationship between them, which may lead to the lack of reliability of the results. Therefore, this paper aims to use propensity score matching (PSM) and select an appropriate instrumental variable (IV) to identify the causal relationship and net impact effect between Internet use and the social network of the elderly to provide a more solid and reliable empirical basis for the study of the impact of Internet use on the social network of the elderly.

## 2. Literature Review 

### 2.1. The Concept and Measurement of Social Network

As early as the 1940s, British anthropologist Radcliffe-Brown first put forward the concept of “social network”, describing the social structure as an actual “network”, and the connection between “points” in the “network” constitutes a way for people to cooperate and communicate [15]. Subsequently, Coleman formally proposed in 1990 that social network is an essential manifestation of social capital for the first time. Individuals can obtain information, resources, and support through social networks and improve individual or collective interests [16]. For individuals, a social network is a collection of people and relationships involved in people’s interpersonal relationships [17]. Research about social networks can be divided into two different research contexts: overall network research and individual network research, but most studies focus on individuals to discuss personal social networks [18]. This paper also pays attention to the social networks of the elderly at the individual level.

Personal social networks are mainly measured by retrospection, association, and nomination methods in the West. For example, the General Social Survey (GSS) in the United States uses the nomination method to require respondents to write down the names of up to five confidants whom respondents reported discussing important issues with them in the past six months, which is further used to determine the core network size, non-kinship, and socio-economic diversity of respondents [19,20]. In addition, different measurement methods such as “friend network”, “core discussion network”, “support network”, and “communication network” are also used to measure personal social networks [17,21]. Some scholars use scales to measure individuals’ social networks, such as Lubben’s Social Network Scale, which measures the differences in the degree of support perceived by individuals from family and friends to measure the status of individuals’ social support networks [22]. 

The measurement methods mentioned above have been applied in China. However, due to the significant differences between Chinese and Western cultures, the measurement of Chinese social networks is also different from that of the West. The essential structure of Chinese society is a differential circle interpersonal network with family as the core, spreading from near to far and extending from layer to layer. Based on this, Chinese Researchers put forward the “Chinese New-Year Greeters’ Networks” with Chinese characteristics, measured by indicators such as the social network’s size, density, potential difference, and upper reachability [23]. In addition, under China’s social and cultural background, a set of social network capital measurement models composed of the size, upper reachability, potential difference, density, and heterogeneity of social networks also was put forward by Wang [18]. Since then, most studies have borrowed the social network capital measurement model proposed by Wang, but different studies have different measurements of social networks. For example, Liu and Ji studied the social network of the elderly in one-child families from two aspects, social network size and structure [24]; Zhang pays attention to the impact of the social network size of the elderly on the quality of life of the elderly [25].

This paper considers that from the perspective of content, the network size can well reflect the extensiveness of individual social networks, while the upper reachability and heterogeneity of social networks can reflect the richness of personal social networks; From the perspective of structure, the size of social network can reflect the horizontal diversity of individual social networks, while the upper reachability and heterogeneity of social network reflect the vertical diversity of social networks. Therefore, in this paper, the social network of the elderly is divided into three aspects: size, heterogeneity, and upper reachability.

### 2.2. Internet Use and the Social Network of the Elderly

There are abundant studies on the influence of Internet use on the social network of the elderly in western academic circles, which have formed two main viewpoints: positive impact and negative effect.

The view of positive influence is “Internet Benefits Theory”. Scholars who agree with this view believe that interpersonal communication relying on the Internet has the advantages of being free from time and space constraints, convenient and efficient, reducing the cost of information communication within social networks [26], and is a convenient and effective means to maintain existing social relations or establish new ties [27]. The use of the Internet expands the social network of the elderly, helps the elderly gradually form a new social interaction mode combining online and offline, improves their social integration level and reduces social isolation [9,11]. Research using data from the American Health and Retirement Study (HRS) found that Internet use increases the frequency of communication between the elderly and others, reconnects the elderly with the outside world [10], and can help the elderly maintain contact with existing friends, make new friends and expand social networks [28]. A study in China also found that communication and interaction between middle-aged and older adults who use short video applications with their family and friends have increased, and their social networks have expanded [29]. 

On the contrary, the view of negative influence is “Replace Theory”. According to this view, the use of the Internet may erode the presence space of the elderly, reduce the real social connection of the elderly, and then harm their social network. This view stems from the “Internet Social Paradox” put forward by Kraut et al., which points out that the more people use the Internet, the less time they use to communicate with their families, the more the social scale is reduced, and the more substantial depression and loneliness they have [12]. Subsequently, the survey using a time log proved the “Replace Effect” and found that there is an apparent substitution relationship between the time spent on the Internet and the time spent on actual social activities, especially the time spent on face-to-face activities with friends and family, which harms individual social networks [13]. The data analysis from the General Social Survey (GSS) in the United States found that mobile phones and the Internet are important reasons people become more isolated, and their core social networks are shrinking [20]. A study using the follow-up survey data of the elderly over 65 years old in the Netherlands also found that Internet use replaced face-to-face communication to a certain extent, reducing the actual community participation of the elderly, the social circle and the friend network of the elderly [14].

### 2.3. Internet Use, Social Participation, and the Social Network of the Elderly

As one of the core connotations of active aging, social participation plays a vital role in improving the social integration of the elderly, meeting their spiritual needs, enhancing their sense of self-belonging, and realizing their value and pursuit. However, due to involuntary reasons such as retirement and disability increases with age, the social participation of the elderly will also gradually decline with the increase in age, the original social network and social connection will slowly disappear, and the social network will shrink [30].

Activity Theory holds that the elderly needs to establish new social relations through active social participation to adapt to the interruption of their original social roles and associations [31]. As a new means of interpersonal communication and socialization, the beneficial effect of Internet use on the social participation of the elderly has been confirmed by many studies [8,32]. Social Capital Theory holds that social participation, as an important way for the elderly to obtain social resources and improve their social status, will expand the social network of the elderly, and further enhance their social support level. Active social participation can enrich the spiritual and cultural life of the elderly, alleviate the psychological obstacles and problems brought about by the changes in living conditions such as retirement and physical function decline, and help the elderly reposition their social roles and form a new way of life. However, negative social participation will lead to further shrinkage of the social network of the elderly and increase the risk of social isolation for the elderly. Based on the above analysis, this study considers that Internet use will expand the social network of the elderly by improving the social participation level of the elderly. In other words, social participation may play a mediating role in the influence of Internet use on the social networks of the elderly.

## 3. Materials and Methods

### 3.1. Data

The data used in this paper are from the Chinese General Social Survey in 2017 (CGSS 2017), organized by the China Survey and Data Center of Renmin University of China. The Chinese General Social Survey is the first nationwide, comprehensive, continuous, and nationally representative large-scale social survey project in China, covering 31 provincial administrative units in the Chinese mainland. CGSS 2017 is the latest survey data released by this project, and 12,582 valid questionnaires were collected in 2017. Module A and Module C of CGSS 2017 data contain the problem of residents’ Internet use, which is a highly representative individual Internet use database in China. Based on the specific Chinese cultural context and the definition of the elderly in the previous research [25,33], the elderly aged 60 or above were selected as the research objective. The samples with missing and invalid values in critical variables such as Internet use and social network were eliminated. Finally, 1363 respondents aged 60 or above were chosen for the analysis.

### 3.2. Variables and Operationalization

#### 3.2.1. Dependent Variable

The social network is the core dependent variable. A social network is a multi-dimensional concept. Combined with the accessibility of data and drawing lessons from existing research [18,33], this paper analyzes the social networks of the elderly from three dimensions: the size, heterogeneity, and upper reachability of social networks.

The social network size of the elderly refers to the number of members in the network. The size of the network determines the number of resources and support that the elderly can absorb from the network. The larger the social network size is, the wider the individual’s social relations and the more resources and support they can obtain from the social network. According to the practice of existing research [33], the scale of the network is measured by the question, “Normally, on a working day, how many people do you contact in a day, whether you know these people or not (contact ways include face-to-face/telephone/internet/chat with other communication devices, conversation, sending messages, etc.)?” In this paper, the answers are divided into five intervals, including “0–4 people”, “5–10 people”, “10–19 people”, “20–49 people”, and “50 people and above”, with values of 1–5, respectively, to measure the social network size of the elderly.

The social network heterogeneity of the elderly refers to the difference in the social network of the elderly. Members’ knowledge structure and background in a low heterogeneity network are similar, and new social resources are limited by mutual communication. The greater the social network heterogeneity is, the richer the social resources it will contain, and the more social resources can be obtained through complementary advantages. Based on previous studies, the number of occupational types included in a personal social communication network is used to measure the social network heterogeneity [34]. The questionnaire asked the respondents, “Are there any family members, relatives, friends, and people you had dealings with before who engaged in the following ten occupations?” The ten occupations are bus/truck driver; Senior managers of large enterprises; Home or office cleaner; Hairdresser; HR Manager; Lawyer; Automobile maintenance worker; Nurses; Police (including traffic police, patrol police, etc.); Junior high school teacher. Assign a value of 1 if there is one and 0 if there is no one. Then, the scores of 10 occupations are added up to get the total number of occupation types involved in the elderly’s communication population to measure the social network heterogeneity of the elderly.

The upper reachability of the social network of the elderly refers to the members at the highest level in the social network of the elderly. The higher the upper reachability of social networks means people with great power, high status, more wealth, and prominent prestige in the social network, which also contains more social resources. Use the question in the questionnaire, “Do you know (know the name and can contact) anybody who engages in the following occupations?”, which also gives ten occupations (the same as above). Referring to existing research [33,35], these ten occupations are divided into five categories, that is, manual workers, service personnel and skilled workers, conventional non-manual workers and self-employed, low-level managers and low-level technicians, and senior managers and senior technicians, with values assigned to 1–5, respectively, and the upper reachability of social network of the elderly is measured by calculating the highest value of the member classes in the social network in which the elderly have.

#### 3.2.2. Independent Variable

The independent variable in this article is internet use. Adopted the question “How often do you use the Internet in the past year?” to reflect the Internet use of respondents, referring to previous research practices [8,33], this paper divides the Internet use of the elderly into two categories: have not used the Internet and used the Internet. Specifically, this paper assigns “never” to 0, that is, “have not used the Internet”, and sets the combination of other options to 1, that is, “used Internet”.

#### 3.2.3. Mediating Variable

The mediating variable in this article is social participation. Social participation includes political participation, leisure participation, cultural inheritance, voluntary service, etc. Due to the limitation of data, this paper pays attention to the overall social participation level of the elderly. The questionnaire of CGSS 2017 asked the elderly about the frequency of participating in three types of activities: “activities organized by leisure groups, sports groups or cultural groups”, “activities organized by political parties, political groups or political organizations”, and “voluntary activities organized by charitable organizations or religious organizations”. Choose from “never participated” to “once a week or more” and assign values of 0–4, respectively. In this paper, the frequency of participating in the three activities is summed up to form an index of the overall social participation level of the elderly, with a value range of 0–12. The higher the score, the higher level of social participation the elderly have.

#### 3.2.4. Control Variable

Referring to the existing research, this paper selects the characteristic factors of the elderly at three levels: individual, family and region as control variables. Among them, individual characteristics include gender, age, education level, marital status, hukou type, religious belief, political status, personal annual income, number of children and self-rated health; Family characteristics include family size and annual income; Regional features include different areas of different provinces. It can be seen from Table 1 that the average age of samples is 69.29; the proportion of older adults using the Internet is 24.1%; the social participation score of the elderly is 1.004. The social network size score is 1.728, the social network heterogeneity score is 2.680, and the upper reachability of the social network score is 2.982. The basic information of variables is shown in Table 1.

### 3.3. Model Construction

#### 3.3.1. The Basic Model

Since the dependent variable social network size, social network heterogeneity, and social network upper reachability are continuous variables, this paper uses the OLS regression model for analysis. The model is set as follows:

(1) $$Y_{i}\;=\beta_{i}\;+\alpha_{i}{\text{internet}}_{i}\;+\gamma_{i}{\text{control}}_{i}\;+\theta_{i}{\text{region}}_{i}+\mu$$

In Equation (1), $Y_{i}$ represents the social network size, social network heterogeneity and social network upper reachability of the elderly. Control means the individual and family characteristic variables controlled in this paper. Region represents regional control variables, $\alpha_{i}$ represents regression coefficients of Internet use, $\beta_{i}$ represents intercept items, $\gamma_{i}$ expresses regression coefficients of individual and family-related control variables, $\theta_{i}$ represents regression coefficients of regional control variables and μ represents residual items.

#### 3.3.2. Propensity Score Matching Model

The Internet use of the elderly is the choice made by the elderly according to their situation, which may have the problem of selective deviation. Therefore, this study further uses the PSM method to deal with the selection deviation of samples and explores the net effect of Internet use on the social network of the elderly. In this study, the Average effect of the Treatment on the Treated (ATT) of the social network of the elderly was estimated through the matching group with the elderly according to whether they use the Internet or not. The specific model is as follows:

(2) $$Y_{i}\;=Y_{0i}\;+(Y_{1i}-Y_{0i})D_{i}$$

(3) $$\mathrm{ATT}=E\;(Y_{1i}-Y_{0i}\;|D_{i}=1)\;$$

In Equation (2), $D_{i}$ is the processing variable, $D_{i}$ = {0,1} indicates the Internet use of the elderly. When $D_{i}$ = 1, it means that the elderly uses the Internet; when $D_{i}$ = 0, it means that the elderly does not use the Internet. In Equation (3), ATT represents the net effect of Internet use on the social network of the elderly.

#### 3.3.3. Mediating Effect Model

This paper also examines the mechanism of the impact of Internet use on the social networks of the elderly, that is, to test the mediating effect of social participation on the relationship between Internet use and social network. We use the mediating effect test steps proposed by Wen [36]. The corresponding model is as follows:

(4) $$Y_{i}\;=\alpha_{i}{\text{internet}}_{i\;}+\mu$$

(5) $$M\;=\beta_{i}{\text{internet}}_{i}\;+\mu$$

(6) $$Y_{i}\;=\theta_{i}{\text{internet}}_{i}\;+\gamma_{i}M\;+\mu$$

In Equations (4) and (6), $Y_{i}$ represents the social network size, social network heterogeneity and social network upper reachability of the elderly. In Equations (5) and (6), *M* represents the social participation of the elderly, $\alpha_{i}$ and $\theta_{i}$ represent effecting coefficients of Internet use on the social network of the elderly, $\beta_{i}$ and $\gamma_{i}$ represent effecting coefficients of Internet use on the social participation of the elderly. Additionally, in (4)–(6) μ represents residual items.

### 3.4. Endogenous Problems

Although the OLS regression model in the last part can preliminarily verify the relationship between Internet use and the social network of the elderly, there may be endogenous problems. First of all, in the OLS regression analysis model, the impact of Internet use on the social network of the elderly may be affected by other confusing variables and then affect the authenticity of the results, so it is difficult to explore the natural effect between Internet use and social network. Secondly, whether to use the Internet is based on the independent choice of the elderly, which will be affected by other factors, not random, and there may be errors in self-selection effect. Finally, due to the limitation of data, some critical control variables related to social networks may be missed. Therefore, based on the existing studies [37], this study takes the Internet connectivity of the family as a tool variable for individual Internet use to deal with endogenous problems. The basis for choosing this index is as follows: First, the network connectivity of the family is an essential prerequisite for the elderly to use the Internet, which will have a more significant impact on the elderly’s use of the Internet and conform to the correlation hypothesis. Second, the Internet connectivity of family as a high-level exogenous environmental variable cannot directly affect the social network of the elderly, which accords with the exogenous hypothesis.

## 4. Results

### 4.1. OLS Regression Analysis

Table 2 shows the OLS regression analysis results of the influence of Internet use on the size, heterogeneity, and the upper reachability of the social networks of the elderly.

In Model 1, the influence of Internet use on the social network size of the elderly was investigated. After controlling the related variables, the regression results show that Internet use significantly expanded the social network size of the elderly (b = 0.277, *p* < 0.01). In Model 2, the influence of Internet use on social network heterogeneity was investigated. After controlling the related variables, the regression results show that Internet use significantly increased the social network heterogeneity of the elderly (b = 1.206, *p* < 0.01). In Model 3, Internet use was used as the independent variable to investigate the influence of Internet use on the upper reachability of social networks of the elderly. After controlling the related variables, the regression results show that Internet use significantly improved the upper reachability of the social network of the elderly (b = 0.619, *p* < 0.01). 

In terms of control variables, the older the elderly are, the smaller their social network size will be; the elderly who are literate, with the higher upper reachability of social network; the elderly with political status as Communist, with more extensive social network size, more significant social network heterogeneity, and higher upper reachability of social network; for the elderly who are engaged in non-agricultural work and have better self-rated health, the larger their social network size will be; the elderly in urban areas are better with more significant social network heterogeneity; the social network of the elderly with more annual family income, with more extensive social network size, more significant social network heterogeneity, and higher upper reachability of social network; Compared with the elderly in the western region, the social network size of the elderly in the central area is more prominent.

### 4.2. Propensity Score Matching

Under the counterfactual framework, the PSM can better deal with self-selection errors by controlling “confusing variables” and helping researchers put forward causal conclusions [38]. To better deal with self-selection errors and model estimation errors caused by confusing variables and enhance the robustness of research results, this paper adopts three different matching methods: K nearest neighbor matching in caliper, radius matching and kernel matching to explore the net effect of Internet use on the social networks of the elderly.

Before PSM, it is necessary to test the balance of samples. As shown in Table 3, before matching, the control variables were significantly different between the treatment and control groups. However, the normalized deviation of most variables after checking is less than 10%, and the difference between the treatment and control groups becomes insignificant. The samples have passed the balance test, which eliminates the imbalance between the control variables, indicating that the PSM can eliminate or reduce the self-selection error of samples to a great extent.

Table 4 shows the average treatment effect (ATT) of the impact of Internet use on the social networks of the elderly under different matching methods. Because there may be errors in the single match results, this paper adopts the self-sampling Bootstrap method to obtain standard errors. It can be seen from Table 4 that the ATT obtained by various matching methods is significant at the statistical level of 5%, that is, after eliminating the sample self-selection error between the treatment group and the control group, Internet use still has a significant positive impact on the social networks of the elderly. Compared with the elderly who do not use the Internet, the elderly who use the Internet score 0.271–0.281 higher on the social network size, 0.921–1.000 higher on the social network heterogeneity and 0.482–0.536 higher on the social network upper reachability. ATT values under different matching methods are close, confirming the robustness of the above research results. At the same time, it also shows that if the study does not deal with the self-selection error in the benchmark regression model, the impact of Internet use on the social networks of the elderly will be overestimated.

### 4.3. Endogeneity Test

The results in Table 5 show that the influence coefficient of tool variables on Internet use of the elderly is 0.319, which is significant at the statistical level of 1%, indicating that tool variables are highly correlated. The F value of the first stage is 198.759, which is much higher than the F critical value of 16.38 in the weak instrumental test at the significance level of 10%, indicating that there is no weak instrumental variable problem. The endogenous test shows that Internet use and social network are endogenous at the statistical level of 1%. The estimation results of the two stages show that after correcting endogeneity, the influence effects of Internet use on the size, heterogeneity, and upper reachability of the social network of the elderly are all significant at the statistical level of 1%, which fully confirms the reliability of the above analysis results. Internet use can indeed benefit the social network of the elderly.

At the same time, after using the instrumental variable method, the influence coefficient of Internet use on the size, heterogeneity, and upper reachability of the social network of the elderly becomes more extensive, which indicates that the influence effect of the Internet on the social networks of the elderly is underestimated due to the existence of endogenous problems.

### 4.4. Mediation Effect Analysis

This paper selects the social participation of the elderly as a mediating variable and analyzes the mechanism of Internet use affecting the social network of the elderly. To test the significance of mediating pathway, the Bootstrap method (repeated sampling 5000 times, confidence interval 95%) is used to estimate the mediating effect. If the 95% confidence interval of mediating effect does not contain 0, it indicates that mediating impact is significant. As shown in Table 6, Internet use has a substantial direct impact on the size, heterogeneity, and upper reachability of the social network of the elderly. The indirect effects of social participation are also significant; social participation plays a crucial mediating role in the influence of Internet use on the size, heterogeneity, and upper reachability of the social network of the elderly (0.026, 0.206 and 0.087, respectively). Internet use will directly affect the social network of the elderly and indirectly affect the social network of the elderly by improving the level of social participation of the elderly.

### 4.5. Different Group Analysis

The relationship between Internet use and the social networks of the elderly may be different among different elderly groups. Since gender, hukou and age are the essential individual attributes, this paper explores the influence of Internet use on the social networks of the elderly of different gender, ages and hukou types, and the results are shown in Table 7.

In terms of gender groups, the effect of Internet use on the heterogeneity and upper reachability of the social network of male elderly is greater than that of female elderly. Still, its influence on the social network size of male elderly is less than that of female elderly. In terms of age groups, the impact of Internet use on the size, heterogeneity, and upper reachability of the social network of the young elderly is far better than that of the middle-aged and older elderly. In contrast, Internet use only significantly impacts the network’s upper reachability of the young elderly. In terms of different hukou types of groups, Internet use has a more substantial effect on the social network heterogeneity of the rural elderly than the city elderly. In contrast, Internet use only has a significant impact on the social network size of the city’s elderly.

## 5. Discussion

Based on the data of CGSS 2017, this paper analyzes the influence of Internet use on the size, heterogeneity, and upper reachability of the social network of the elderly. It explores the influence mechanism of Internet use on the social networks of the elderly, which further deepens the research on the relationship between Internet use and the social network of the elderly. The main conclusions of the study are as follows:

First, Internet use has a significant positive impact on the size, heterogeneity, and upper reachability of the social network of the elderly. At the same time, this paper further proves that the positive impact of Internet use on the social network of the elderly has high robustness by using the PSM method and IV method under the condition of eliminating or reducing the self-selection problem of samples and solving endogenous problems. This is consistent with the conclusions of existing research [10,26], which confirms the “Internet Benefits Theory”, indicating that in the Chinese context, Internet use also has a significant positive impact on the social networks of the elderly in China. Moreover, a recent study by Chinese researchers also found that Internet use significantly reduced loneliness levels of older Chinese adults [39]. Collectively, these results suggest that there may be a complex relationship between Internet use, loneliness, and the social network of elderly people, which needs to be explored further.

Second, social participation plays an important mediating role between Internet use and the social networks of the elderly. Internet use will benefit the social network of the elderly by improving their social participation level, further expanding the size and heterogeneity of the social networks of the elderly and improving the social network’s upper reachability of the elderly. Activity theory holds that the elderly can realize new social participation and gain new social roles using Internet technology, which can alleviate the problems of shrinking social networks and rising risk of social isolation caused by the interruption of social functions in old age and help the elderly to re-recognize themselves, thus improving their social network.

Third, the influence of Internet use on the social network of the elderly with different genders, ages and hukou types is heterogeneous. In terms of gender groups, the effect of Internet use on the heterogeneity and upper reachability of the social network of male elderly is greater than that of female elderly. Still, its influence on the social network size of male elderly is less than that of female elderly. The possible reason is that the social network size of male older adults is larger [4], and the beneficial effect of Internet use on their social network size tends to weaken due to the diminishing marginal effect. In terms of age groups, the impact of Internet use on the size, heterogeneity, and upper reachability of the social network of the young elderly is far better than that of the middle-aged and older elderly. In contrast, the impact on the upper reachability of the social network of the middle-aged and older elderly is not significant. It may be because, with the increase in age, middle-aged and older elderly are more inclined to keep social contact with people who are relatively close [40] and less keen to make friends with people with higher social status. Additionally, some research revealed that the social network of the elderly was subject to urban-rural differences [41]. We also found that Internet use has a more significant impact on the social network heterogeneity of the rural elderly than the urban elderly. In contrast, Internet use only has a considerable effect on the social network size of the urban elderly. Compared with the urban elderly, the social network of the rural elderly is characterized by high convergence, low heterogeneity, and small social network size [42,43]. This may be the main reason why Internet use, a non-institutional factor, can play a more significant role in the social network of the elderly in rural areas than in urban areas. However, the rural areas are an acquaintance-oriented society, and the elderly have lived together for generations. Their social network size is relatively stable, and the Internet has little influence on them. Still, it will increase the heterogeneity of their social groups and upgrade their upper reachability of the social network.

Fourthly, this study also finds that age, engaging in non-agricultural work, self-rated health, and annual household income has a significant impact on the social network of the elderly. The older the elderly are, the smaller their social network size will be, consistent with the core viewpoint of the Socioemotional Selectivity Theory and Social Convoy Model. With the increase in age, the social network of the elderly gradually shrinks [30,40]. However, the elderly engaged in non-agricultural work and have good self-rated health have a larger social network size. Consistent with the existing research, the social network size of healthy older adults is larger [4]. Engaging in non-agricultural work is an important way for the elderly to re-socialize, which can help them expand the size of their social networks. The better the annual household income is, the better the living conditions and environment are, and the more resources individuals can obtain, with more heterogeneity of social networks and higher upper reachability of the social network. The social network of the elderly with political status as a communist with larger size and heterogeneity of social network and higher upper reachability of the social network. Communist membership is a social status indicator with Chinese characteristics. In China, Communist membership is not only a critical reference condition for applying to be a civil servant and other occupations, but also the recruitment process of Communist members will comprehensively consider the social status of applicants [44], so party memberships can also affect individual social networks to a certain extent.

### 5.1. Implications

These findings have significant implications for formulating public policies. Under the background of the information society, the Internet, as an important choice for the elderly to connect with their families, peers, and the whole community, plays an increasingly important role in maintaining or expanding the social network of the elderly. Therefore, it is necessary for relevant departments to take adequate measures to bridge the “digital divide”. There are several realistic achievable measures that can be enacted to bridge the “digital divide”.

First, the government can make more investment into further improving Internet infrastructure construction in rural areas and providing more convenient infrastructure conditions for rural elderly to gain access to the Internet since the use of the Internet has beneficial effects on the social network of both rural and urban elderly. Secondly, relevant departments should do an excellent job in developing applications that are suitable for the elderly, ensuring the applications meet the actual needs of the elderly groups and helping them use the Internet more comfortably. Thirdly, communities and family members should give timely help and guidance and encourage the elderly to use the Internet. This will gradually resolve the difficulties existing in the process of Internet use of the elderly and improve the elderly’s confidence. Fourthly, it is also necessary to help the elderly establish the concept of lifelong learning and provide Internet Safety and Usage Education for the elderly and provide more training and guidance on Internet use skills for the elderly so that the elderly can better master basic Internet skills and improve their digital literacy.

### 5.2. Limitations

There are still some deficiencies in this paper as follows. First, because the size and proportion of the elderly using the Internet in China are still relatively low with relatively simple use, this paper mainly adopts the primary condition of whether to use the Internet when measuring Internet use. With the further optimization of the “aging-suitable” Internet, the Internet use of the elderly will become increasingly diversified. In the future, the impact of different Internet uses of the elderly on the social network of the elderly could be considered in future studies. Secondly, the connotation of social networks is extremely rich. Limited by the data, this paper exploratively investigates the impact of Internet use on the social network of the elderly from three aspects: the size, heterogeneity, and upper reachability of the social network. The different impacts of Internet use on other social network types of the elderly can be deeply explored in future studies, for example, the social network of friends or family. Thirdly, the latest publications identify the fact that Internet use significantly reduces loneliness among older Chinese adults [39]. It is also of great significance to analyze the relationship between internet use, loneliness, and social network; however, due to the data limitations, this study could not investigate the relationship between them, which we will continue to explore in the future.

## 6. Conclusions

Internet use has a profound impact on the social participation and social networks of the elderly. In China, Internet use expands the social network size, enlarges the social network heterogeneity, and enhances the social network upper reachability of the elderly. These results also passed the robustness test. There is heterogeneity in the effect of Internet use on social networks among the elderly of different genders, ages, and places of residence. The impact of Internet use on the heterogeneity and upper reachability of the social network of male elderly is more significant than that of female elderly. Still, its influence on the social network size of male elderly is less than that of female elderly. The impact of Internet use on the size, heterogeneity, and upper reachability of the social network of the 60–69-year-old elderly is far greater than that of the 70-years and over elderly. However, the impact on the upper reachability of the social network of the 70-years and over elderly is not significant. Internet use has a more significant impact on the social network heterogeneity of the rural elderly than the city elderly. In contrast, Internet use only has a considerable effect on the social network size of the city’s elderly. Social participation plays a critical mediating role in the influence of Internet use on the social networks of the elderly. Internet use will not only benefit the social network of the elderly directly, but will improve their social participation to exert a positive influence on the social network of the elderly indirectly. Therefore, it is of great importance to utilize the Internet to enhance the level of social participation of the elderly and reshape their social network of the elderly. The government must pay more attention to the beneficial effect of Internet use on the elderly’s social networks and take adequate measures to bridge the “digital divide” and promote the digital integration of the elderly.

## Figures and Tables

**Table 1:** Descriptive statistical results of samples.

Variable		Mean/%	SD
Dependent variable			
Social network size		1.728	0.968
Social network heterogeneity		2.680	2.649
Social network upper reachability		2.982	1.996
Independent variable			
Internet use		0.241	0.428
Mediating variable			
Social participation		1.004	1.976
Control variable			
Gender (%)	Men	48.79	
	Women	51.21	
Age		69.29	7.432
Marital status (%)	Have spouse	73.59	
	No spouse	26.41	
Education (%)	Illiteracy	24.06	
	Literacy	75.94	
Religious relief (%)	Irreligion	88.41	
	Profess a religion	11.59	
Political status (%)	Communist	15.26	
	Noncommunist	84.74	
Individual annual income (ln)		8.183	3.488
Self-rated health		3.012	1.111
Non-agricultural work (%)	Engaged	7.70	
	Not engaged	92.30	
Living style (%)	Living alone	20.85	
	Living with others	79.15	
Number of children		2.430	1.537
Type of hukou (%)	City	40.94	
	Rural	59.06	
Family size		2.505	1.649
Annual household income (ln)		9.770	2.379
Region (%)	Western	21.50	
	Central	32.36	
	Eastern	46.15	

**Table 2:** Regression analysis of the influence of Internet use on the social networks of the elderly.

	Social Network Size	Social Network Heterogeneity	Social Network Upper Reachability
Internet use	0.277 ***	1.206 ***	0.619 ***
	(0.072)	(0.200)	(0.150)
Gender (reference: women)	0.005	0.161	0.059
	(0.057)	(0.158)	(0.118)
Age	−0.012 ***	−0.009	−0.002
	(0.004)	(0.012)	(0.009)
Marital status (reference: no spouse)	0.101	−0.021	−0.004
	(0.083)	(0.230)	(0.172)
Education (reference: illiteracy)	0.054	0.261	0.258 *
	(0.071)	(0.196)	(0.146)
Religious belief (reference: irreligion)	−0.021	−0.246	−0.207
	(0.090)	(0.248)	(0.186)
Political status (reference: noncommunist)	0.240 ***	0.425 *	0.534 ***
	(0.080)	(0.220)	(0.165)
Individual annual income	0.014	0.004	0.032
	(0.010)	(0.027)	(0.020)
Self-rated health	0.084 ***	0.092	0.064
	(0.025)	(0.070)	(0.053)
Non-agricultural work (reference: not engaged)	0.515 ***	0.439	0.321
	(0.101)	(0.280)	(0.210)
Living style (reference: living alone)	0.141	0.042	−0.084
	(0.090)	(0.248)	(0.186)
Number of children	0.027	−0.058	0.010
	(0.020)	(0.056)	(0.042)
Type of hukou (reference: rural)	−0.014	0.506 ***	0.228
	(0.068)	(0.187)	(0.140)
Family size	0.022	−0.010	0.007
	(0.018)	(0.050)	(0.038)
Annual household income	0.028 *	0.069 *	0.052 *
	(0.015)	(0.040)	(0.030)
Region (Western)			
Central	0.155 **	0.280	0.141
	(0.074)	(0.203)	(0.152)
Eastern	−0.006	−0.257	0.190
	(0.079)	(0.217)	(0.163)
N	1363	1363	1363
R^2^	0.122	0.110	0.113

Noted: *** *p* < 0.01, ** *p* < 0.05, * *p* < 0.1.

**Table 3:** Sample balance test.

Variable	Matching Situation	Treatment Group	Control Group	Deviation (%)	Deviation Reduction (%)	T	*p*
Gender	Unmatching	0.574	0.478	19.4	82.4	2.87	0.004
	Matching	0.566	0.583	−3.4		−0.41	0.685
Age	Unmatching	66.965	69.916	−42.2	98.6	−6.01	0.000
	Matching	67.168	67.128	0.6		0.07	0.941
Marital status	Unmatching	0.858	0.714	35.6	96.3	4.96	0.000
	Matching	0.853	0.848	1.3		0.18	0.859
Education	Unmatching	0.972	0.700	79.0	98.4	9.89	0.000
	Matching	0.971	0.967	1.3		0.31	0.760
Religious belief	Unmatching	0.080	0.104	−8.5	49.1	−1.23	0.220
	Matching	0.082	0.070	4.3		0.56	0.577
Political status	Unmatching	0.311	0.101	53.8	73.6	8.99	0.000
	Matching	0.290	0.346	−14.2		−1.41	0.159
Individual annual income	Unmatching	10.129	7.636	85.5	93.1	11.30	0.000
	Matching	10.075	9.904	5.9		1.01	0.311
Self-rated Health	Unmatching	3.346	2.931	39.1	98.5	5.67	0.000
	Matching	3.326	3.320	0.6		0.08	0.940
Non-agricultural work	Unmatching	0.152	0.058	31.2	87.4	5.23	0.000
	Matching	0.136	0.148	−3.9		−0.40	0.687
Living style	Unmatching	0.145	0.227	−21.1	72.5	−2.99	0.003
	Matching	0.151	0.128	5.8		0.76	0.446
Number of children	Unmatching	1.595	2.65	−78.1	95.9	−10.71	0.000
	Matching	1.613	1.656	−3.2		−0.50	0.619
Type of hukou	Unmatching	0.889	0.491	95.4	97.1	12.77	0.000
	Matching	0.885	0.874	2.8		0.42	0.673
Family size	Unmatching	2.426	2.455	−1.8	−345.0	−0.27	0.788
	Matching	2.423	2.554	−8.0		−1.04	0.299
Annual household income	Unmatching	11.094	9.385	90.2	99.0	11.50	0.000
	Matching	11.038	11.056	−0.9		−0.18	0.856
Region	Unmatching	1.367	1.882	−70.6	90.8	−10.10	0.000
	Matching	1.370	1.322	6.5		0.89	0.375

Noted: In the table, K nearest neighbor matching method in the caliper is adopted (K = 4), and other test results have passed the balance test.

**Table 4:** ATT values of Internet use on the social networks of the elderly.

Variables	Matching Method	ATT	Bootstrap SE	T
Social network size	K nearest neighbor matching in caliper	0.281	0.125	2.60 **
	Radius matching	0.271	0.102	2.79 ***
	Kernel matching	0.271	0.110	2.79 ***
Social network heterogeneity	K nearest neighbor matching in caliper	1.000	0.326	3.48 ***
	Radius matching	0.921	0.278	3.51 ***
	Kernel matching	0.928	0.279	3.53 ***
Social network upper reachability	K nearest neighbor matching in caliper	0.536	0.241	2.67 ***
	Radius matching	0.482	0.204	2.53 **
	Kernel matching	0.487	0.196	2.55 **

Noted: K nearest neighbor matching in caliper (K = 4, caliper value = 0.05); Radius matching caliper value = 0.05; Kernel matching uses the default kernel function and bandwidth; Bootstrap sampling is set to 1000 times; *** *p* < 0.01, ** *p* < 0.05; SE means standard errors.

**Table 5:** Regression results of the instrumental variable.

Variable	One-Stage Internet Use	Two-Stage Social Network Size	Two-Stage Social Network Heterogeneity	Two-Stage Social Network Upper Reachability
Internet use		0.620 *** (0.189)	1.689 *** (0.522)	1.295 *** (0.392)
Instrumental variable	0.319 *** (0.023)			
Control variable	YES	YES	YES	YES
One-stage F value	198.759			
Endogenous test *p*-value	0.000			

Noted: *** *p* < 0.01.

**Table 6:** Bootstrap mediation test results.

Action Path	Effect	Coefficient	Bootstrap SE	LLCI	ULCI
Internet use-Social network size	Direct effect	0.237	0.080	0.082	0.394
	Indirect effect	0.026	0.014	0.003	0.059
Internet use-Social network heterogeneity	Direct effect	0.960	0.213	0.554	1.377
	Indirect effect	0.206	0.065	0.102	0.357
Internet use-Social networks upper reachability	Direct effect	0.530	0.146	0.257	0.820
	Indirect effect	0.087	0.029	0.040	0.156

Noted: SE means standard errors; LLCI and ULCI represents the Lower and the Upper 95% confidence interval respectively.

**Table 7:** Analysis of the influence of Internet use on the social network of the different elderly groups.

Variable	Gender		Age		Type of Hukou	
	Male	Female	60–69	70 and over	City	Rural
Social network size	0.221 ** (0.108)	0.344 *** (0.062)	0.293 *** (0.095)	0.252 ** (0.117)	0.306 *** (0.088)	0.214 (0.162)
Social network heterogeneity	1.625 *** (0.278)	0.760 *** (0.293)	1.442 *** (0.258)	0.664 ** (0.331)	1.196 *** (0.246)	2.138 *** (0.424)
Social network upper reachability	0.831 *** (0.206)	0.495 ** (0.222)	0.911 *** (0.187)	0.110 (0.259)	0.684 *** (0.171)	0.721 * (0.370)

Noted: *** *p* < 0.01, ** *p* < 0.05, * *p* < 0.1.

## Data Availability

The data were released to the researchers without access to any personal data. Data access link: http://cgss.ruc.edu.cn/ (accessed on 27 November 2020).
